# Acetate and Potassium Modulate the Stationary-Phase Activation of *lrgAB* in *Streptococcus mutans*

**DOI:** 10.3389/fmicb.2020.00401

**Published:** 2020-03-13

**Authors:** Sang-Joon Ahn, Shailja Desai, Loraine Blanco, Min Lin, Kelly C. Rice

**Affiliations:** ^1^Department of Oral Biology, College of Dentistry, University of Florida, Gainesville, FL, United States; ^2^Department of Biology, College of Liberal Arts and Sciences, University of Florida, Gainesville, FL, United States; ^3^Department of Chemistry, College of Liberal Arts and Sciences, University of Florida, Gainesville, FL, United States; ^4^Department of Microbiology and Cell Science, Institute of Food and Agricultural Sciences, University of Florida, Gainesville, FL, United States

**Keywords:** *Streptococcus mutans*, pyruvate, acetate, potassium, LrgAB

## Abstract

Fluctuating environments force bacteria to constantly adapt and optimize the uptake of substrates to maintain cellular and nutritional homeostasis. Our recent findings revealed that LrgAB functions as a pyruvate uptake system in *Streptococcus mutans*, and its activity is modulated in response to glucose and oxygen levels. Here, we show that the composition of the growth medium dramatically influences the magnitude and pattern of *lrgAB* activation. Specifically, tryptone (T) medium does not provide a preferred environment for stationary phase *lrgAB* activation, which is independent of external pyruvate concentration. The addition of pyruvate to T medium can elicit P*lrgA* activation during exponential growth, enabling the cell to utilize external pyruvate for improvement of cell growth. Through comparison of the medium composition and a series of GFP quantification assays for measurement of P*lrgA* activation, we found that acetate and potassium (K^+^) play important roles in eliciting P*lrgA* activation at stationary phase. Of note, supplementation of pooled human saliva to T medium induced *lrgAB* expression at stationary phase and in response to pyruvate, suggesting that LrgAB is likely functional in the oral cavity. High concentrations of acetate inhibit cell growth, while high concentrations of K^+^ negatively regulate *lrgAB* activation. qPCR analysis also revealed that growth in T medium (acetate/K^+^ limited) significantly affects the expression of genes related to the catabolic pathways of pyruvate, including the Pta/AckA pathway (acetate metabolism). Lastly, stationary phase *lrgAB* expression is not activated when *S. mutans* is cultured in T medium, even in a strain that overexpresses *lytST*. Taken together, these data suggest that *lrgAB* activation and pyruvate uptake in *S. mutans* are connected to acetate metabolism and potassium uptake systems, important for cellular and energy homeostasis. They also suggest that these factors need to be implemented when planning metabolic experiments and analyzing data in *S. mutans* studies that may be sensitive to stationary growth phase.

## Introduction

LrgAB homologs were recently reported to function as a pyruvate transporter in *Bacillus subtilis* (Charbonnier et al., 2017; van den Esker et al., 2017), and most recently in *Streptococcus mutans* (Ahn et al., 2019), a primary causative agent of human dental caries. Although pyruvate has a large potential to modulate various virulence traits via cell homeostasis for improved survival and persistence of various bacteria (Busuioc et al., 2010; Chaudhari et al., 2016; Hawver et al., 2016; Vilhena et al., 2018, 2019), to date little is known about the role and regulation of pyruvate in these organisms. However, interest in pyruvate transporters and metabolism is growing, primarily as a result of increasing evidence for their roles in biological fitness and resuscitation in bacterial communities (Hawver et al., 2016; Vilhena et al., 2019). Such roles of pyruvate may be especially important to *S. mutans*, given its ability to withstand the limited resources and environmental fluctuations experienced in the oral cavity, and during pathogenic biofilm development on the tooth surface. Pyruvate is excreted as an overflow metabolite (Paczia et al., 2012), thus a common nutrient in microbiome environments such as the oral cavity (Takahashi et al., 2010; Gawron et al., 2019). When bacterial cells experience nutrient limitation (during the transition to stationary phase), they rapidly initiate re-uptake of previously excreted pyruvate through LrgAB (Paczia et al., 2012; Ahn et al., 2019). Consistent with the observation of stationary phase uptake of pyruvate, we recently found that although supplemented pyruvate had no impact on the growth rate of *S. mutans* cells, it did prolong the exponential phase of growth (Ahn et al., 2019), presumably enabling cells to take up pyruvate according to their needs and ensuring long-term survival. Along with other α-keto acids (i.e., α-ketoglutarate, oxaloacetate), pyruvate is also known to effectively scavenge ROS (Reactive oxygen species), including hydrogen peroxide (H_2_O_2_), through a non-enzymatic oxidative decarboxylation mechanism (Constantopoulos and Barranger, 1984; O’Donnell-Tormey et al., 1987; Desagher et al., 1997; Mizunoe et al., 1999). We recently demonstrated that stationary phase *lrgAB* induction is modulated by H_2_O_2_ and by co-cultivation with the H_2_O_2_-producing oral commensal, *Streptococcus gordonii* (Ahn et al., 2019), buffering external sources of oxidative stress. Also, of note, the reaction of pyruvate with H_2_O_2_ produces water, CO_2_, and acetate (CH_3_-CO-COOH + H_2_O_2_ → CH_3_-COOH + H_2_O + CO_2)_ (Giandomenico et al., 1997), and acetate has also reported to be taken up into bacterial cells in parallel with pyruvate under nutrient limited growth conditions (Jolkver et al., 2009; Paczia et al., 2012), as well as to cause cell death in *S. aureus* (Sadykov et al., 2013; Thomas et al., 2014; Chaudhari et al., 2016). Therefore, these observations further support a potential role for pyruvate as a signal to initiate a metabolic response to deal with nutrient-limited conditions, which may modulate homeostasis and virulence of bacteria.

From a *S. mutans* metabolic standpoint, pyruvate is produced from glucose through glycolysis, and forms a node among a partial TCA (tricarboxylic acid) cycle, fatty acid biosynthesis, and biosynthesis of amino acids, such as leucine. These occur via acetyl-CoA which is converted from pyruvate by the Pdh (pyruvate dehydrogenase) complex or Pfl (pyruvate formate lyase), depending on the presence or absence of oxygen, or the limitation or excess abundance of a preferred sugar (e.g., glucose) (Abbe et al., 1991; Colby and Russell, 1997). Pyruvate can be also fed to three different organic acid production pathways, leading to excretion of lactate, acetate, and formate. The production of this mixture of organic acids in an appropriate ratio is beneficial to individual bacteria by maintaining cellular redox balance (NAD^+^/NADH) and maximizing the ATP yield per glucose to promote cell homeostasis. Due to its central role in metabolism, Pdh is known to be regulated at both the biochemical and genetic levels in *Escherichia coli* (Hansen and Henning, 1966; Schwartz and Reed, 1970; Shen and Atkinson, 1970; Datta, 1991). In *S. mutans*, the genes encoding the Pdh complex are also dramatically upregulated in response to carbohydrate depletion, an expression pattern that is shared with *lrgAB*, encoding a pyruvate uptake system (Busuioc et al., 2010; Ahn et al., 2019).

It is interesting that stationary phase *lrgAB* expression increases within 1 h, and the level of accumulated pyruvate appears to be the primary stimulus for *lrgA*B induction and subsequent pyruvate uptake (Kim et al., 2018; Ahn et al., 2019), suggesting that the metabolic status of the cell and environment may play a key role in pyruvate regulation. Since expression of *lrgAB* and activity of LrgAB is modulated by external glucose and oxygen levels (Ahn et al., 2010, 2019), pyruvate regulation seems to be coordinated by environmental fluctuations. In this study, we show the effect of specific growth medium constituents on exponential phase and stationary phase *lrgAB* responses, particularly the involvement of acetate and potassium (K^+^) in coordinating the expression of *lrgAB* and the uptake of pyruvate. These findings further suggest that LrgAB activity and pyruvate uptake can be modulated by both environmental and metabolic conditions.

## Materials and Methods

### Bacterial Strains and Growth Conditions

*Streptococcus mutans* UA159 and its derivative strains were grown in brain heart infusion (BHI) medium (Difco) as overnight static cultures at 37°C in a 5% CO_2_ atmosphere. Antibiotics were used to supplement growth media in the following concentrations: erythromycin (10 μg/ml), kanamycin (1 mg/ml), and spectinomycin (1 mg/ml). The media used include BHI, chemically defined medium FMC (Terleckyj and Shockman, 1975), T (Tryptone), TV (Tryptone/Vitamin), TY (Tryptone/Yeast extract), and TVY (Tryptone/Vitamin/Yeast extract) (Burne et al., 1987, 1999). All media were supplemented by 11 mM glucose (named FMC11, T11, TV11, TY11, and TVY11, respectively), except for BHI. For growth measurements, fresh medium was inoculated with 1:100 dilutions of overnight cultures of *S. mutans*. Each medium was supplemented by sodium pyruvate (Na-pyruvate; Fisher Scientific), as indicated for each experiment. The optical density at 600 nm (OD _600_) was measured at 37°C at 30 min-intervals using a Bioscreen C growth curve analysis system. At least three independent experiments, each in quadruplicate, were performed. A representative result is presented in each relevant figure.

### Microplate Reporter Assay

GFP intensity of *S. mutans* strains harboring a P*lrgA-gfp* reporter fusion, previously constructed (Lauderdale et al., 2010; Son et al., 2012, 2015; Kim et al., 2018), were observed using a Synergy microplate reader (BioTek) controlled by Gen5 software (Kaspar et al., 2017, 2018; Kim et al., 2018). Overnight cultures of the reporter strains were diluted 1:50 into 2 ml of BHI broth, grown to an OD_600_ ≈ 0.5, and then diluted 1:50 into 175 μl fresh media, including BHI, FMC11, T11, TV11, TY11, and TVY11, in individual wells of a 96-well plate (black walls, clear bottoms; Corning). Media were supplemented by potassium chloride (KCl; Sigma-Aldrich), potassium phosphate (K-phosphate; Fisher Scientific), sodium pyruvate, sodium acetate (Na-acetate; Sigma-Aldrich), sodium chloride (NaCl; Fisher Scientific), and sodium phosphate (Na-phosphate; Fisher Scientific), as indicated for each experiment. This reporter assay was also performed in TV11, supplemented by commercial pooled human saliva (Innovative Research, MI). Saliva was used after filter sterilization through a 0.22 μm filter. The OD_600_ and green fluorescence were monitored (sensitivity = 45; excitation = 485 nm; emission = 520 nm) at 30 min intervals. The fluorescence of wildtype harboring plasmid without the reporter gene fusion was subtracted from fluorescence readings of *S. mutans* strains harboring the P*lrgA-gfp* gene fusion. At least three independent replicates, each in triplicate, were performed. A representative result is presented in each relevant figure.

### Construction of *lytST*-Overexpressing Strain

A strain constitutively expressing *lytST* was constructed as previously described (Ahn and Rice, 2016). Briefly, we first generated a fragment (ΩKm-P*ldh*) containing a polar kanamycin resistance gene (ΩKm) and a *ldh* promoter region (P*ldh*), replacing the promoter region of *lytS* (P*lytS*). Two ∼0.45-kb fragments flanking the −35 and −10 sequences of the *lytS* promoter were PCR-amplified, ligated into the ΩKm-P*ldh* cassette, and transformed into *S. mutans*. Transformants were selected on BHI agar containing kanamycin, and double-crossover recombination into each gene was confirmed by PCR and sequencing to ensure that no mutations were introduced into flanking genes. This *lytST*-overexpressing strain (SAB163) was then transformed by the P*lrgA-gfp* construct for GFP quantification assay.

### Measurement of Extracellular Pyruvate Levels

*Streptococcus mutans* UA159 wild-type strain was grown in BHI, TV11 or TY11 medium. For time course measurements of extracellular pyruvate during growth, samples (250 μl) were taken at 1–2 h intervals and 100 μl was used to measure the OD_600_ in a spectrophotometer for monitoring growth. The rest of volume (150 μl) was centrifuged for 2 min at 18,000 g to remove the cells, and pyruvate concentration of the supernatant were quantified with an EnzyChrom^TM^ pyruvate assay kit (BioAssay Systems, Hayward, CA, United States), according to the manufacturer’s instructions. The results are average or representative of two independent replicates, each performed in duplicate.

### Quantitative Real-Time PCR (qPCR) Assay

To measure the expression of genes using qPCR, *S. mutans* UA159 was grown in BHI, FMC11 and TV11 broth at 37°C in a 5% (vol/vol) CO_2_ atmosphere. Cells was also grown in TV11, supplemented by 10 mM KCl, to verify the effect of potassium on stationary phase *lrgAB* induction. To measure growth-dependent expression of genes related to pyruvate metabolism, cells were harvested in early-exponential and -stationary growth phases. Extraction of RNA, qPCR, and data analysis were performed as described elsewhere (Ahn et al., 2012; Ahn and Rice, 2016; Rice et al., 2017). Expression was normalized against an internal standard (*gyrA*). Statistical analyses were performed on data generated from *n* = 3 independent experiments using an unpaired *t*-test.

## Results

### The Magnitude of P*lrgA* Activation and Pyruvate Flux in Response to Stationary Phase Depends on the Growth Media

**FIGURE 1:**
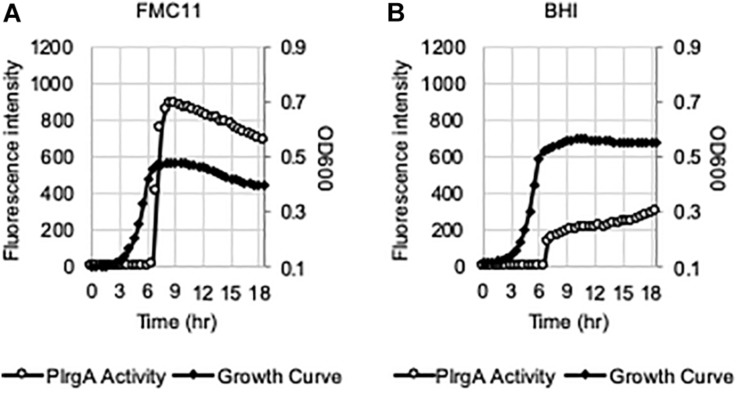
P*lrgA* activation in both chemically-defined FMC11 and rich complex BHI media. The P*lrgA-gfp* reporter strain was grown in FMC medium, supplemented by 11 mM glucose (FMC11, **(A)**, and BHI (brain heart infusion, **(B)**. Relative *gfp* expression (gray circle) and cell growth (OD_600_; black diamond) were monitored during growth on a plate reader (see section “Materials and Methods” for details). The results are representative of five independent experiments.

**FIGURE 2:**
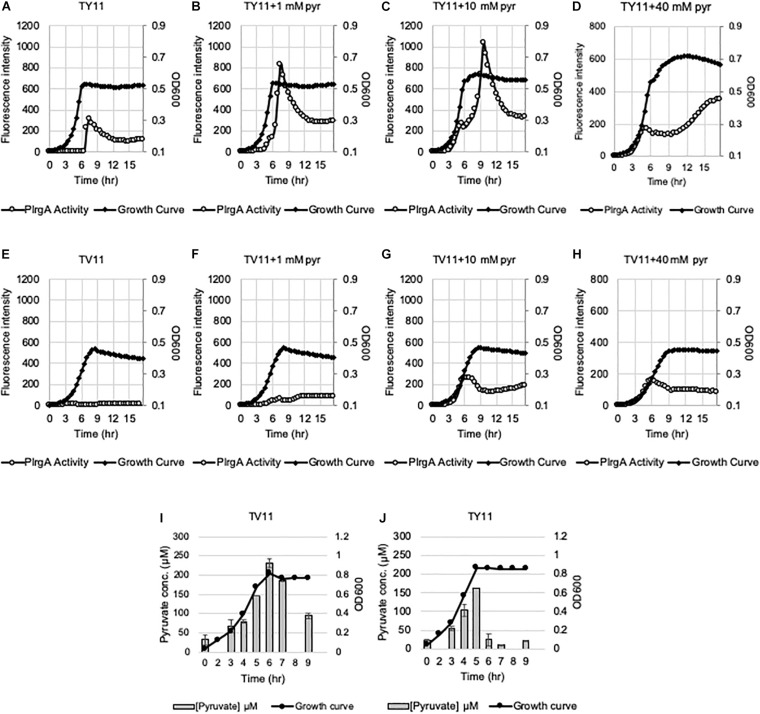
Change of P*lrgA* activity and extracellular pyruvate during growth in TY11 and TV11 media, supplemented by different concentrations of extracellular pyruvate. For measurement of P*lrgA* activation, the P*lrgA-gfp* reporter strain was grown in a low (11 mM)-glucose TY (TY11, **A–D**) and TV (TV11, **E–H**) media, supplemented by 0 **(A,E)**, 1 **(B,F)**, 10 **(C,G)**, 40 mM **(D,H)** pyruvate (pyr). Relative *gfp* expression (gray circle) and cell growth (OD_600_; black diamond) were monitored during growth on a plate reader (see section “Materials and Methods” for details). The results are representative of three independent experiments. For measurement of extracellular pyruvate, *S. mutans* wild type UA159 was grown in TV11 **(I)** and TY11 **(J)**. For time course measurements of extracellular pyruvate and growth, samples were taken at 1 or 2 h intervals (see section “Materials and Methods” for details). The concentration of pyruvate was determined using an EnzyChrom pyruvate assay kit, and cell growth was measured by the optical density at 600 nm (OD_600_). Bars indicates the average concentration of extracellular pyruvate; solid line with circles indicates the corresponding growth curve. The results are average of two independent experiments. Error bars = standard deviation.

We have recently shown that the *lrgAB* promoter (P*lrgA*) is activated in cells which face glucose depletion (stationary-phase) and sense the presence of extracellular pyruvate, typically secreted as an overflow metabolite during exponential growth (Ahn et al., 2019). For measurement of P*lrgA* activation, we utilized a fluorescent strain, previously created by fusing the P*lrgA* to *gfp* in a shuttle vector and introduced the construct into *S. mutans* wild-type UA159 (UA159/P*lrgA-gfp*) (Kim et al., 2018; Ahn et al., 2019). The fluorescence intensity of green fluorescent protein (GFP) to quantify P*lrgA* activation was measured in cultures grown in FMC medium (Terleckyj et al., 1975), because a rich medium, such as BHI, generates a very large autofluorescence background. i.e., the fluorescence intensity in plain BHI is about 3-fold higher than that in plain FMC. However, we noticed that when we measured the response of P*lrgA* during growth in BHI, stationary phase induction of *lrgAB* was about 75% reduced (Figure 1B), compared to that observed in FMC11 (Figure 1A). In both media, containing the same glucose level (11 mM), cells reached the onset of stationary phase in 6.5 h, in which glucose seems to be exhausted, as reported previously (Ahn et al., 2019). Nevertheless, the final yield was higher in BHI (OD_600_ = 0.56) than FMC11 (OD_600_ = 0.47), suggesting that the primary carbohydrate was more actively metabolized for cell growth than other metabolic pathways and cellular processes in BHI. The maximum level (∼80 μM) of pyruvate excreted during growth in BHI medium was also about 80% reduced (Supplementary Figure S1), compared to that (∼400 μM) observed in FMC11 (Ahn et al., 2019), supporting the previously-observed pyruvate concentration dependence of P*lrgA* activation. Since both BHI and FMC11 media had the same glucose content (11 mM), the lower-than expected pyruvate excretion in BHI, compared to that in FMC11, could reflect other significant differences between the compositions of these two media which may affect P*lrgA* activation and pyruvate flux. To further evaluate dependence of P*lrgA* activation and pyruvate flux on media composition, we also monitored the response of P*lrgA* over growth in two different tryptone-based media, TY (**T**ryptone/**Y**east extract) and TV (**T**ryptone/**V**itamin mix) media (Burne et al., 1987, 1999), both used extensively for *S. mutans* studies. Both TY and TV media were supplemented by 11 mM glucose (named TY11 and TV11, respectively), equivalent to the concentration found in BHI and FMC11 media. Growth in TY11 still generated a sharp P*lrgA* signal upon entry to stationary growth phase (Figure 2A), as observed in both FMC11 and BHI cultures (Figures 1A,B). However, the maximum level of P*lrgA* activation in TY11 (Figure 2A) was about 25% lower than in FMC11 (Figure 1A) but higher than in BHI (Figure 1B). Another difference in TY11 is that the activated P*lrgA* level rapidly declined at the beginning of stationary phase (Figure 2A), suggesting that *S. mutans* cells may experience a metabolic shift different from that which occurs in FMC11. In contrast, when the reporter strain was cultivated in TV11, stationary phase P*lrgA* activation was almost undetectable (Figure 2E). A similar inhibitory response on P*lrgA* was also observed when the strain was cultivated in T11 medium, lacking vitamin mix (Supplementary Figure S2A), indicating that stationary phase repression of P*lrgA* was not due to the vitamin mix in TV11. Notably, when we added 0.3% yeast extract (equivalent to that used in TY11) into TV11 (named TVY11) and cultivated the reporter strain, the response of P*lrgA* to stationary phase was restored to comparable levels as observed in TY11 (Supplementary Figure S2E), indicating that growth in tryptone alone as a peptide source provides an unsuitable environment for stationary phase response of P*lrgA* that requires unknown constituent(s) found in yeast extract, as well as FMC and BHI. Given the correlation between overflowed pyruvate levels and P*lrgA* activation (Ahn et al., 2019), we also measured the extracellular concentration of pyruvate during cultivation of the *S. mutans* wild-type strain in TV11 and TY11. As shown in Figure 2I, the TV11 culture excreted levels (∼225 μM) of pyruvate about 44% less to that observed in FMC11 (∼400 μM) when peak levels of pyruvate are observed (Ahn et al., 2019). The re-uptake of pyruvate was also markedly decelerated and over 40% of excreted pyruvate still remained in TV11 medium until 3 h after the onset of stationary phase (Figure 2I). Even in TY11 culture, the peak level (∼160 μM) of pyruvate excreted during growth was about 60% reduced, compared to that previously observed in FMC11, but pyruvate concentrations were rapidly depleted (Figure 2J), similar as previously observed in FMC11 culture (Ahn et al., 2019). Thus, these results suggest that P*lrgA* activation is not dependent on the excreted pyruvate concentration only, and can be blocked by additional factor(s) or mechanism(s) present in TV11 cultures.

### The Response of P*lrgA* to External Pyruvate Is Reduced When the Cell Is Cultured in Tryptone

**FIGURE 3:**
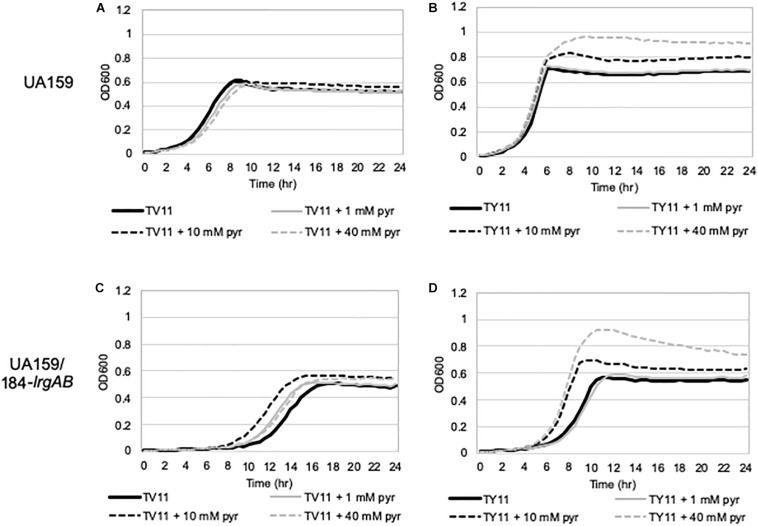
The effect of exogenously added pyruvate on the growth of *S. mutans* wild-type and *lrgAB*-overexpressing strains. Wild type **(A,B)** and *lrgAB*-overexpressing (UA159/184-*lrgAB*; **C,D**) strains were grown in TV11 **(A,C)** and TY11 **(B,D)**, supplemented by different concentrations of pyruvate (0, 1, 10, and 40 mM). Optical density at 600 nm was monitored every 30 min at 37°C using the Bioscreen C lab system. The results are representative of three independent experiments.

To further explore the blockage of P*lrgA* activation in TV11, we assumed that excreted pyruvate may be unable to normally stimulate P*lrgA* in TV11 cultures. Thus, we tested three different pyruvate concentrations to evaluate whether P*lrgA* activation still depends on external pyruvate concentrations in TV11 cultures, as previously observed in FMC11, showing that supplementation of 1 mM exogenous pyruvate to FMC11 at time of inoculation elevates the *lrgAB* induction level by about 3-fold (Ahn et al., 2019). In that study, the degree of P*lrgA* activation increased linearly with increasing concentrations of pyruvate up to 10 mM, and further increase of supplemented pyruvate led to a decrease of P*lrgA* activation (Ahn et al., 2019), presumably due to a negative feedback regulation acting on LytST by the presence of high levels of extracellular pyruvate (Charbonnier et al., 2017; Ahn et al., 2019). However, the response of P*lrgA* to 1 mM (Figure 2F), 10 mM (Figure 2G), and 40 mM (Figure 2H) exogenous pyruvate in TV11 was not remarkable, only showing a moderate increase at stationary phase. This result suggests that the response of P*lrgA* to external pyruvate may be interfered with in TV11 cultures. More interestingly, supplying exogenous pyruvate to TV11 led to the activation of P*lrgA* during exponential phase (Figures 2F–H), implying that extracellular pyruvate may be taken up and consumed by *S. mutans* cells even before glucose is depleted in TV11. The early activation of P*lrgA* was most evident in the presence of 10 mM pyruvate (Figure 2G) compared to 1 mM pyruvate (Figure 2F). However, no further activation was observed in the presence of 40 mM pyruvate (Figure 2H). A similar trend was also observed in T11 medium, supplemented by pyruvate (Supplementary Figures S2A–D), further supporting that the vitamin mix in TV11 has no influence on the response of P*lrgA* to extracellular pyruvate. When we repeated this experiment in TY11, which allowed a moderate and short activation of P*lrgA* (Figure 2A), the same early activation of P*lrgA* was observed with supplementation of 1 mM (Figure 2B), 10 mM (Figure 2C), and 40 mM (Figure 2D) pyruvate. However, the stationary phase activation of P*lrgA* also increased with supplementation of 1 mM and 10 mM pyruvate, although it did not seem to occur in a dose-dependent manner, as was observed in FMC11 (Ahn et al., 2019). Further increase of supplemented pyruvate (40 mM) led to a drastic decrease of P*lrgA* activation, presumably due to negative feedback regulation (Ahn et al., 2019). These observations suggest that growth in tryptone-based media may perturb fluxes in central carbohydrate metabolism in a different way from that observed in FMC11, subsequently modulating the timing to take up external pyruvate. We also observed that high levels (10 mM and 40 mM) of external pyruvate prolongs the exponential growth of *S. mutans* in TY11 (Figures 2C,D) but not in TV11 (Figures 2G,H), in accordance with the P*lrgA* activation data. Again, this trend was also observed in TVY11, supplemented by extracellular pyruvate (Supplementary Figures S2E–H), further supporting that the stationary-phase response of P*lrgA* can be enhanced by a constituent(s) included in yeast extract. To verify these read-outs, we monitored growth of the wild-type strain in TV11 and TY11, supplemented with increasing amounts of pyruvate (0, 1, 10 and 40 mM) using a Bioscreen C plate reader. As expected, supplementation of pyruvate to TV11 had no clear effect on further cell growth at stationary phase (Figure 3A), while supplemented pyruvate prolonged the exponential phase of growth in a dose-dependent manner in TY11 (Figure 3B). No obvious change was observed during exponential growth in the same conditions, although P*lrgA* was activated during exponential phase (Figures 2B–D,F–H). To determine if this phenotype was due to insufficient exponential phase activation of P*lrgA*, we monitored growth of a strain overexpressing *lrgAB* (UA159/184-*lrgAB*) (Ahn and Rice, 2016), in this same condition. The addition of external pyruvate to TV11 (Figure 3C) and TY11 (Figure 3D) markedly enhanced the growth rate of the *lrgAB* overexpression strain. However, no further growth enhancement was observed at stationary phase in TV11 (Figure 3C), while the addition of external pyruvate to TY11 effectively prolonged exponential growth (Figure 3D). In BHI, added pyruvate had no impact on growth rate of the *lrgAB*-overexpressing strain but still effectively prolonged exponential growth in a dose-dependent manner (Supplementary Figure S3). Taken together, these results suggest that external pyruvate can be taken up and consumed as a carbon source even during exponential growth (or in the presence of excess glucose) when the cell is grown in tryptone-based media. They also suggest that the uptake and utilization of external pyruvate for further cell growth at stationary phase may be elicited by a constituent(s) that is absent in tryptone but present in yeast extract, as well as that may be required for the activation of LytST-LrgAB circuit.

### Acetate Contributes to the Response of P*lrgA* to Stationary Phase

**FIGURE 4:**
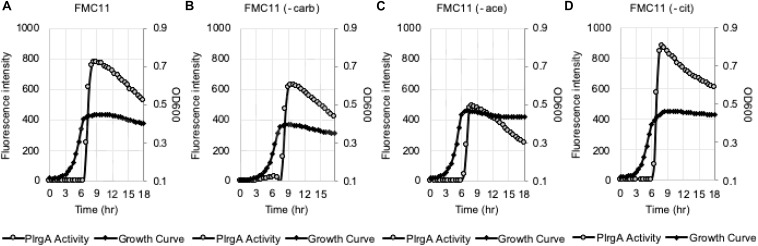
The effect of the major compositions of FMC medium on P*lrgA* activation. The P*lrgA-gfp* reporter strain was grown in a complete FMC11 **(A)** and FMC11, lacking carbonate (-carb, **B**), acetate (-ace, **C**), or citrate (-cit, **D**). Relative *gfp* expression (gray circle) and cell growth (OD_600_; black diamond) were monitored during growth on a plate reader (see section “Materials and Methods” for details). The results are representative of three independent experiments.

**FIGURE 5:**
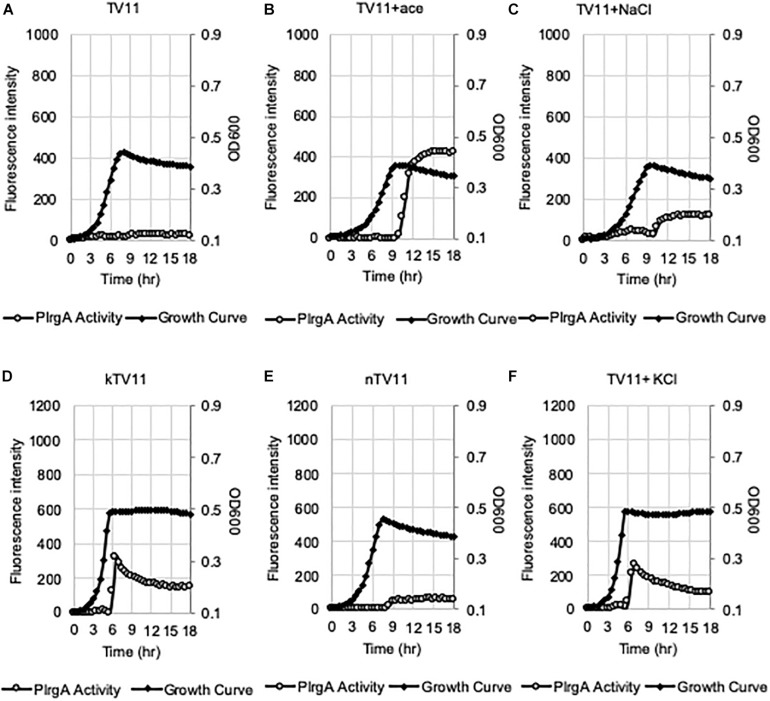
Evaluation of the potential elicitors for the activation of P*lrgA* in TV11. The P*lrgA-gfp* reporter strain was grown in TV11 **(A)** and TV11, supplemented by 73.14 mM acetate (ace, **B**), 73.14 mM NaCl **(C)**, 10 mM K-phosphate (kTV11, **D**), 10 mM Na-phosphate (nTV11, **E**), and 10 mM KCl **(F)**. Relative *gfp* expression (gray circle) and cell growth (OD_600_; black diamond) were monitored during growth on a plate reader (see section “Materials and Methods” for details). The results are representative of three independent experiments.

We assumed that the potential medium constituent(s), mediating stationary phase P*lrgA* activation, may be most abundant in FMC11, relative to BHI and TY11, but lacking in TV11. Thus, we set out to test the effect of several major components of FMC on P*lrgA* activation by removing each component from FMC, using the same *gfp* reporter strain. FMC contains an assortment of amino acids, vitamins, and metals, required for normal cell growth, as well as Na-acetate (73.14 mM), Na-citrate (7.65 mM), and Na-carbonate (12.8 mM) as bulk growth components (Terleckyj et al., 1975). Here, we evaluated whether Na-acetate, Na-citrate, or Na-carbonate contributes to P*lrgA* activation at stationary phase, because they are likely included in FMC at higher concentrations than those in tryptone. Na-carbonate is known to increase growth yield of *S. mutans*, due to its increased buffering capacity and an active role of CO_2_ fixation in the metabolism of the organism (Terleckyj et al., 1975). As shown in Figure 4B, when the reporter strain was cultivated in FMC11, lacking Na-carbonate, both growth yield and response of P*lrgA* to stationary phase moderately decreased, compared to that observed in the compete FMC11 (Figure 4A). After normalization by cell growth (OD_600_), the P*lrgA* activity was less than 10% reduced relative to that in FMC (data not shown). The final pH was also similar in both cultures (data not shown). Thus, it suggests that the contribution of Na-carbonate to P*lrgA* activation is not major. Interestingly, when the reporter strain was cultivated in FMC11, lacking Na-acetate, the growth was slightly enhanced but the stationary phase response of *PlrgA* was more than 35% reduced (Figure 4C), compared to that in the complete FMC (Figure 4A), indicating that Na-acetate contributes to P*lrgA* activation. To verify the effect of Na-acetate on P*lrgA* activation, we also cultivated the reporter strain in TV11, unfavorable for P*lrgA* activation, supplemented by 73.14 mM Na-acetate (the same concentration as in FMC) and monitored the response of P*lrgA* throughout growth. Figure 5A confirms that P*lrgA* is not activated in TV11 (Figure 2E). The addition of Na-acetate into TV11 markedly triggered P*lrgA* activation up to about 55% level of that observed in the complete FMC (Figures 4A, 5B). The addition of 73.14 mM Na-acetate to TV11 has no significant effect on pH (data not shown). The high concentration of sodium (Na^+^) in Na-acetate does not seem to be a major contributor to P*lrgA* activation, although the addition of sodium chloride (NaCl; 73.14 mM) to TV11 had a slight effect on P*lrgA* activation (Figure 5C). Therefore, these results suggest that the acetate, included in FMC at a high concentration, substantially contributed to P*lrgA* activation, particularly at stationary phase. As shown in Figure 5B, high concentration of acetate inhibited growth, extending lag-phase and lowering the final yield, in accordance with the observation for growth enhancement in FMC11 lacking Na-acetate (Figure 4C). The growth defect conferred by high acetate levels is known to be a metabolic consequence (Smirnova and Oktiabr’skii, 1988; Axe and Bailey, 1995; Pinhal et al., 2019), although its underlying mechanism still remains elusive. Nevertheless, this result suggests that the extracellular concentration of acetate affects P*lrgA* activation. Lastly, when we performed the experiment in FMC11, lacking Na-citrate, we observed that the growth yield and P*lrgA* activation were only slightly enhanced (Figure 4D) relative to complete FMC11 (Figure 4A), suggesting that citrate has no marked effect on P*lrgA* activation. We also observed that the addition of metals (Mg^2+^, Fe^2+^, and Mn^2+^) to TV11 at the concentration equivalent to that included in FMC had no obvious effect on P*lrgA* activation (data not shown), suggesting that traces of metals are not major contributors to P*lrgA* activation.

### Potassium (K^+^) Is Important for P*lrgA* Activation

**FIGURE 6:**
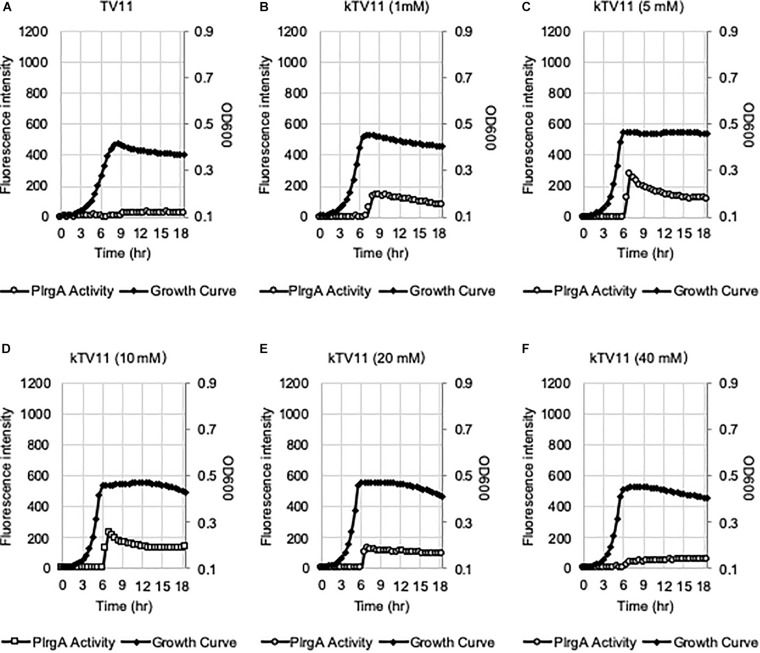
The effect of different concentrations of potassium on eliciting the P*lrgA* activation in TV11. The P*lrgA-gfp* reporter strain was grown in TV11, supplemented by different concentrations of potassium (**A**, 0 mM; **B**, 1 mM; **C**, 5 mM; **D**, 10 mM; **E**, 20 mM; and **F**, 40 mM) as K-phosphate (kTV11). Relative *gfp* expression (gray circle) and cell growth (OD_600_; black diamond) were monitored during growth on a plate reader (see section “Materials and Methods” for details). The results are representative of three independent experiments.

In our recent study, we showed that P*lrgA* activation was significantly inhibited by two hydrophobic protonophores, CCCP (carbonyl cyanide m-chlorophenyl hydrazine) and DNP (2,4-dinitrophenol) (Ahn et al., 2019), suggesting that pH change may have an effect on P*lrgA* activation. In fact, the culture pH varies in different media during exponential phase, and this possibly contributes to the different activation of P*lrgA*. Supplementary Figure S4 shows that the culture pH in FMC11 falls fairly steadily from 7.0 to 5.8 as cells progress into late-exponential phase (Supplementary Figure S4A). In BHI, TY11, and TV11, the pH further falls to 5.6, 5.3, and 5.3, respectively (Supplementary Figures S4B–D). Thus, although an acidic environment does not necessarily impact P*lrgA* activation, it is possible that FMC is more buffered than the others, leading to the highest activation of P*lrgA*. In this regard, FMC contains 10 mM potassium (K^+^)-phosphate as a relatively mild pH buffer (Terleckyj et al., 1975). So, we buffered TV11 with the same concentration of K-phosphate (named kTV11), cultivated the *gfp* reporter strain for measurement of P*lrgA* activation in kTV11, and monitored the GFP intensity over growth. Interestingly, P*lrgA* activation was markedly elicited at stationary phase in this buffered TV (Figure 5D), similar to that in TY11 (Figure 2A). As well, the addition of 1, 10, and 40 mM pyruvate to kTV11 (Supplementary Figures S5B–D) exhibited similar patterns of P*lrgA* activation and prolonged exponential growth to those observed in TY11 (Figures 2B–D). When we monitored the pH change during growth in kTV11, the culture pH fell to 5.3 (Supplementary Figure S4E) that is close to those in TV11 and TY11 (Supplementary Figures S3C,D). This suggests that P*lrgA* activation in kTV11 was not due to pH buffering but possibly to potassium (K^+^). To test this, K-phosphate was replaced by equimolar quantities of sodium (Na)-phosphate in TV11 (named nTV11) and we repeated the experiment. As anticipated, the degree of P*lrgA* activation in nTV11 was markedly reduced (Figure 5E), compared to that in kTV (Figure 5D), although a slight activation of P*lrgA* was observed. The response of P*lrgA* to increasing concentrations of external pyruvate (1, 10, and 40 mM) in nTV11 (Supplementary Figures S5E–H) is much closer to those in TV11 (Figures 2F–H), suggesting that K^+^ was a key player in stationary phase P*lrgA* activation. Also, of note, supplemented pyruvate was utilized for further cell growth at stationary phase in kTV11 (Supplementary Figures S5C,D, S6A), similar to that in TY11 (Figures 2C,D, 3B), but not in nTV11 (Supplementary Figures S5G,H, S6B). To verify the involvement of K^+^ in P*lrgA* activation, the experiment was repeated in TV11, supplemented by 10 mM potassium chloride (KCl). The trend of P*lrgA* activation in TV11 supplemented by KCl (Figure 5F) was similar to that in kTV11 (Figure 5D) and TY11 (Figure 2A). To verify the read-out by GFP fluorescence, we also monitored changes in the expression of *lrgAB* during the transition to stationary phase in TV11 with and without 10 mM KCl, using quantitative real-time PCR (qRT-PCR). As shown in Supplementary Figure S7, the addition of KCl to TV medium led to a greater increase of P*lrgA* activation in stationary phase compared to growth in TV without supplementation, further supporting the results measured by GFP quantification. Therefore, these results strongly suggest that P*lrgA* activity can be modulated in response to K^+^ availability. Furthermore, to assess the K^+^ concentration dependence of P*lrgA* activation, we monitored P*lrgA* activation in TV11 supplemented with increasing amount of K-phosphate (from 1 to 40 mM). Figures 6A,B show that P*lrgA* activation can be elicited by addition of 1 mM K-phosphate to TV11 but does not increase in a dose-dependent manner (Figures 6C–F). Conversely, it declines by addition of high concentrations of K-phosphate (20 and 40 mM; Figures 6E,F). This trend was also observed in FMC11, supplemented by increasing amount of K-phosphate (from 10 to 100 mM; Supplementary Figures S8A–D). The activation level of P*lrgA* decreased as the concentration of K-phosphate increased. This reduction was most drastic between 20 and 50 mM (Supplementary Figures S8C,D). This observation suggests that K^+^ may be required for stationary phase P*lrgA* activation that would be rapidly switched off if cells encounter a sudden increase in the environmental K^+^ concentration. As summarized in Supplementary Figure S9, the data show that high level of P*lrgA* activation in FMC11 is due to acetate and K^+^ that may be scarce or absent in tryptone.

### P*lrgA* Is Activated in the Presence of Saliva

**FIGURE 7:**
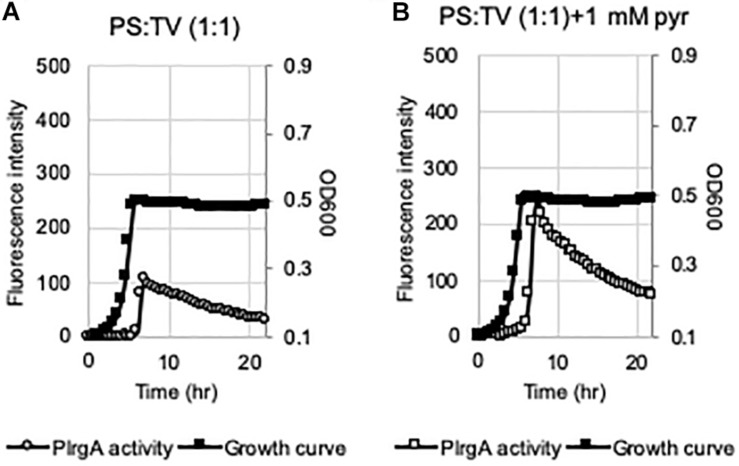
The activation of P*lrgA* in the presence of saliva. The P*lrgA-gfp* reporter strain was grown in TV, supplemented by commercial pooled human saliva (PS) at 1:1 ratio (TV:PS), without **(A)** or with **(B)** pyruvate (1 mM). Relative *gfp* expression (gray circle) and cell growth (OD_600_; black diamond) were monitored during growth on a plate reader (see section “Materials and Methods” for details). The results are representative of two independent experiments.

Given that medium components, such as acetate and K^+^, play an important role in eliciting P*lrgA* activation at stationary phase, we next wondered if *lrgAB* can be actually induced *in vivo*, i.e., in the oral cavity. To explore this, we attempted to monitor the response of P*lrgA* over growth in the presence of pooled human saliva (PS). The P*lrgA*-*gfp* reporter strain was grown in TV (non-inducing medium), supplemented by commercial PS at 1:1 ratio (PS:TV), due to relatively high viscosity of PS. The final glucose content was set to 11 mM. As shown in Figure 7A, the supplementation of PS to TV medium by 50% noticeably elicited P*lrgA* activation at stationary phase. Also, of note, the addition of 1 mM pyruvate to the PS-supplemented TV medium increased the P*lrgA* signal by >2-fold (Figure 7B). Thus, these results suggest that *lrgAB* can be induced and respond to external pyruvate in the presence of saliva which may contain nutrient component(s), including K^+^, required for P*lrgA* activation.

### The Effect of Tryptone on Pyruvate Metabolism

Since the level of acetate, a major by-product of pyruvate, impacts P*lrgA* activation, we decided to monitor changes in the expression of pyruvate catabolism-related genes during the transition to stationary phase in BHI, FMC11 and TV11 cultures, using qRT-PCR. We first confirmed that *lrgAB* was dramatically upregulated at early-stationary phase, compared to early-exponential growth phase by about 32,000-fold in BHI and by about 7,850-fold in FMC11, while no significant change was observed in TV11 (Table 1). The *pdhC* gene, encoding one of the components of the pyruvate dehydrogenase complex (Pdh), was also remarkably upregulated at early-stationary phase, compared to that of early-exponential phase, by about 795-fold in BHI and by about 635-fold in FMC11, in agreement with our previous observation (Ahn et al., 2012, 2019). In contrast, in TV11, *pdhC* was not differentially expressed between the two growth phases (Table 1). In the absence of oxygen, pyruvate can be also converted by pyruvate formate lyase (Pfl) to acetyl-CoA and formate. The expression of *pfl*, encoding Pfl, was also markedly increased in both BHI and FMC11, but not in TV11. Thus, these results indicate that the conversion of pyruvate to acetyl coenzyme (acetyl-CoA) by Pdh and Pfl is strongly correlated with the regulation of *lrgAB.* By this logic, the repression of P*lrgA* at stationary phase in TV11 may result, in part, from inefficient conversion of pyruvate to acetyl-CoA. Acetyl-CoA is further metabolized to acetate with the production of one molecule of ATP by the Pta-AckA pathway (Carlsson et al., 1985). qPCR analysis showed that *pta* was upregulated by about 5.3-fold in BHI and by about 3.7-fold in FMC, respectively, while the expression was still not altered in TV11, as cells approached stationary phase (Table 1). However, *ackA* was moderately downregulated in all media, although the differential expression was not significant in TV11. Thus, increased acetyl-CoA levels by Pdh and Pfl seems to induce the expression of *pta*, encoding phosphate acetyltransferase (Pta), responsible for the conversion of acetyl-CoA to acetyl-P. Nevertheless, the increased level of acetyl-P does not appear to affect the activity of AckA (acetate kinase), responsible for the conversion of acetyl-P to acetate, which may be more tightly regulated, considering that acetyl-P is known to transfer phosphate groups to and acetylate regulatory proteins (Fox et al., 1986; Kim et al., 1996, 2001; Gueriri et al., 2008; Liu et al., 2009; Castano-Cerezo et al., 2014; Kuhn et al., 2014). Similar to *pta*, the expression of *adhE*, encoding acetaldehyde dehydrogenase, responsible for the conversion between acetyl-CoA and acetaldehyde, was markedly upregulated by about 4.7-fold in BHI and by 5.5-fold in FMC11, while down-regulated in TV11, as cells approached stationary phase, further supporting the idea that increased acetyl-CoA levels may elicit its catabolic pathways. Overall, these observations suggest that growth in a low-glucose tryptone medium impacts the catabolism and overflow of pyruvate as the cells approach stationary phase, consequently modulating the need and capacity of the organism to take up external pyruvate. They further suggest that P*lrgA* activation and pyruvate flux are coordinated at both the genetic and metabolic levels in pyruvate pathways.

**TABLE 1:** The comparison of pyruvate metabolism-related gene expression in early-exponential (EE) vs. -stationary (ES) growth phases of *S. mutans* UA159 by real-time qPCR.

	Fold-change (ES/EE)		
	BHI	FMC11	TV11
*lrgA*	7,850.40*	32,004.90*	0.65
*pdhC*	795.72*	635.02*	0.99
*pfl*	27.36*	3.47^+^	0.47
*pta*	5.35*	3.69*	0.65
*ackA*	0.62^+^	0.33*	0.5
*adhE*	4.76*	5.52^+^	0.02*

Statistical analyses were performed using an unpaired t-test. *P ≤ 0.01; ^+^ P ≤ 0.05.

### Repression of *lrg* at Stationary Phase of TV Cultures Is Independent of LytST

**FIGURE 8:**
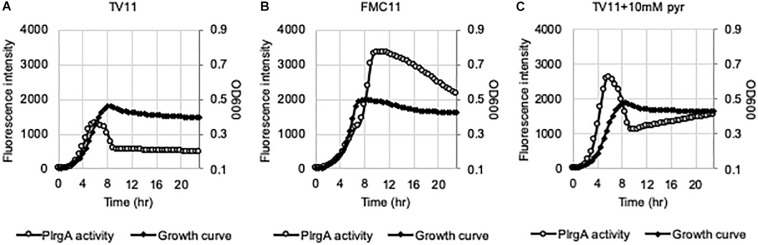
The activation of P*lrgA* in a *lytST*-overexpression strain. The P*lrgA-gfp* reporter strain in a *lytST* overexpressing background (SAB163) was grown in TV11 **(A)**, FMC11 **(B)**, and TV11, supplemented by 10 mM pyruvate (pyr, **C**). Relative *gfp* expression (gray circle) and cell growth (OD_600_; black diamond) were monitored during growth on a plate reader (see section “Materials and Methods” for details). The results are representative of three independent experiments.

Given that *lrgAB* expression requires activation by LytST TCS (Ahn et al., 2010), we wondered if stationary phase repression of *lrgAB* in TV11 cultures occurred through LytST. To explore this idea, we introduced the P*lrgA-gfp* construct into the *lytST*-overexpressing strain (SAB163), in which *lytST* was equally overexpressed under the control of a *ldh* promoter and ribosome binding site (RBS) over growth. We observed that *lytS* was about 10-fold more expressed in SAB163 using real-time qPCR, relative to that in the wild-type (data not shown). Notably, overexpression of *lytST* remarkably relieved the repression of *lrg* during exponential growth in TV11, but stationary phase induction of *lrg* still appeared to be suppressed (Figure 8A). However, when we monitored the expression of *lrg* during growth in FMC11, we observed that the expression of *lrg* was induced during exponential growth, and dramatically elevated at stationary phase (Figure 8B). These results suggest that repression of *lrgAB* during growth may be primarily due to inaccessibility of LytT to the promoter region of *lrgAB*, but stationary phase repression of *lrg* in TV11 cultures occurs independently of LytST. Even when the reporter strain was cultivated in TV11, supplemented by 10 mM pyruvate, stationary phase induction of *lrg* was still not evidently elevated, even though exponential phase induction of *lrg* increased in response to supplementation of pyruvate (Figure 8C). Intriguingly, in FMC11 cultures, we also noticed that there was a short lag phase between late-exponential and stationary phase, suggesting a metabolic/regulatory transition for *lrg* expression (Figure 8B).

## Discussion

The question of how P*lrgA* activation (for pyruvate uptake) is regulated is of importance for understanding the metabolism of *S. mutans*, but may also have implications for therapeutic application to restrict the survival of this organism in a metabolically competitive oral microflora (i.e., during development of plaque biofilms). Here we show that the response of P*lrgA* can be remarkably altered by nutrient concentrations other than glucose, which was previously shown to be critical for P*lrgA* activation (Ahn et al., 2010, 2019; Kim et al., 2018). It is not surprising, because pyruvate is a central carbon metabolite and in flux among several key pathways, responsible for the production of acetyl-phosphate, organic acids and ATP, and the conversion of NAD/NADH. Our new data presented here suggest that the composition of the growth medium modulates the degree and timing of P*lrgA* activation, consequently determining how and when environmental pyruvate is taken up by the cell, which in turn may facilitate cellular adaptation. We tested both complex culture media (BHI, TV and TY) and chemically-defined medium (FMC), all of which are frequently used in *S. mutans* laboratory experiments. It is possible that the alleviated stationary phase response of P*lrgA* observed in complex media, such as BHI and tryptone media, is attributable to accelerated pyruvate consumption in the cell, consequently reducing excretion of pyruvate during growth and subsequent activation of P*lrgA*. Indeed, complex media provides needed precursors for the biosynthesis of macromolecules. A remarkable finding in this study is that stationary phase P*lrgA* activation is very limited in low-glucose tryptone (T) medium (or TV). During growth in T medium, the response of P*lrgA* to external pyruvate does not follow a typical “burst” of expression in stationary-phase. Instead, P*lrgA* appears to be activated during exponential growth in T medium. Our results suggest that bacteria monitor the extracellular environment and may need to sense additional extracellular nutrient(s) or metabolite(s) in order to adapt their transport capacities and metabolism for utilization of alternative carbon source to ensure long-term survival. If identified, these extracellular factor(s) may have potential to limit the survival and persistence of the organism.

It may not be surprising that the level of external acetate has an impact on eliciting P*lrgA* activation at stationary phase, considering that acetate is excreted as an overflow metabolite during growth to eliminate extra redox potential when glucose is no longer oxidized to CO_2_. The accumulation of acetate in the medium inhibits growth, although its underlying mechanism remains elusive. Possibly, acetate may be protonated to acetic acid (diffusible form), which subsequently dissociates into acetate and proton in the cell, as environmental pH approaches the pKa (∼4.8) of acetate. Thus, the excess protons need to be pumped out of the cell in order to maintain the membrane potential. But this process costs ATP and draws away energy from growth (Smirnova and Oktiabr’skii, 1988; Axe and Bailey, 1995). More interestingly, the cytoplasmic acidification by excess acetate has been hypothesized to potentiate cell death in *S. aureus*, which is mediated by *cidBC* (Rice et al., 2005; Thomas et al., 2014), the other components of the *cid/lrg* system (Ahn et al., 2010). Thus, the hypothesized role of LrgAB in induced cell death and lysis may be attributed to changes of environmental acetate and pyruvate levels, overflowed during growth. Considering that acetate is the major by-product of pyruvate reaction with H_2_O_2_, the environmental level of acetate may serve as another metabolic cue to elicit LrgAB as a pyruvate uptake system, facilitating cellular adaptation and competition with H_2_O_2_-producing oral commensals. In fact, acetate is fairly abundant (18–56 mM) in plaque fluids (Margolis and Moreno, 1994), likely sufficient to at least stimulate LrgAB for uptake of pyruvate under a carbohydrate-limited condition. We are currently investigating how LrgAB and environmental pyruvate levels influence the interaction between *S. mutans* and *S. gordonii* as a function of long-term survival and persistence, and whether acetate mediates the functional interplay between Cid and Lrg, postulated in previous studies (Ahn et al., 2010; Ahn and Rice, 2016).

At high concentrations of external acetate, the flux direction of the Pta-AckA pathway, responsible for the conversion of pyruvate to acetate, can perturbate fluxes in central metabolism and allow the cell to consume acetate while growing on glucose (Pinhal et al., 2019). In accordance with this, our real-time qPCR analysis shows that the expression of genes, encoding the key enzymes related to pyruvate catabolism, including the Pta-AckA pathway, are significantly altered during the transition to stationary phase in FMC (acetate enriched) but not in TV (acetate limited). Excess acetate may also change the intracellular concentration of acetyl-P, known to transfer phosphate groups to regulatory proteins, including LytT, responsible for expression of *lrgAB*. In our study, it is unclear whether and how external acetate and pyruvate interplay to maintain metabolic homeostasis, which needs further quantitative characterization of pyruvate/acetate fluxes, as well as energetic variables and membrane potential. Nevertheless, this study uncovers the involvement of acetate in regulating pyruvate uptake through LrgAB, and identifies promising directions in which to further investigate the linkage between pyruvate metabolism and cell death/lysis mechanism.

Another interesting finding is that the activation of P*lrgA* at stationary phase requires potassium (K^+^), essential for cell growth. K^+^ is naturally abundant in all types of cells and present in the range of 41–85 mM in the fluid portion of dental plaque (Dibdin et al., 1986; Margolis and Moreno, 1994). It is not produced in the cell and thus, its acquisition depends on uptake. K^+^ is provided as K-phosphate (10 mM) in FMC and likely included in yeast extract, because supplementation of the same concentrations of K-phosphate or KCl to TV medium elicits P*lrgA* activation in a very similar manner to that observed in TY11. In fact, according to the ingredient table of yeast extract (BD Bionutriuent^TM^ Technical Manual)^1^, yeast extract has 58.0 mg K^+^/g, thus the K^+^ concentration of TY is about 3.7 mM, which seems to be sufficient to elicit P*lrgA* activation in this study. In *S. mutans*, four putative K^+^ transport systems, annotated as Trk1 (*trkB-trk-pacL*; SMU_1561-1563), Trk2 (*trkA-trkH*; SMU_1708-1709), Kch (SMU_1848), and GlnQHMP (SMU_1519-1522) were previously reported by Binepal et al. (2016). In that study, the authors showed that Trk2 is the main player for K^+^ uptake and K^+^-dependent cellular response to environmental stresses in *S. mutans*. As well, other studies have also reported that K^+^ influences glycolysis, membrane potential, and pH homeostasis (Sato et al., 1989; Dashper and Reynolds, 1992). Also of interest, our previous microarray analysis showed that *trkB*, encoding a putative potassium uptake system, and SMU_1848 were upregulated by about 10-fold and 4-fold, respectively, at late-exponential phase, compared to early-exponential growth phase, which is shared with *lrgAB* (Ahn et al., 2012; Kim et al., 2018). Collectively, these observations highlight not only the fact that pyruvate uptake and metabolism are intimately connected to K^+^ uptake and homeostasis, but also the fact that its regulation is linked with major virulence attributes, including stress tolerance, and essential cellular function of *S. mutans*. It is also interesting that the level of P*lrgA* activation is independent of K^+^ concentration, as 20 mM and higher K^+^ alleviates the activation of P*lrgA* without affecting cell growth. Thus, if environmental K^+^ concentration increases, the capability of the cell to take up external pyruvate may decline. Given that K^+^ accumulation can occur under stress conditions (Christian and Waltho, 1961; Csonka, 1989), pyruvate uptake and its subsequent catabolism for energy (ATP) production might be elicited via K^+^ homeostasis process. In addition, in this current study, high levels of K^+^ (up to 100 mM) do not appear to affect the growth of *S. mutans* in TV11 or FMC11, although they had an appreciable influence on the magnitude of cell lysis at stationary phase. This conflicts with a previously-published observation that lower (≤5 mM) or higher (≥25 mM) K^+^ concentrations in minimal defined medium with 1% (55 mM) glucose (MMGK) resulted in growth retardation (Binepal et al., 2016). Although the precise mechanisms require further study, glucose and yet-unknown medium components(s) could affect K^+^, and subsequently pyruvate uptake for cell growth and homeostasis. It is also noteworthy that the addition of saliva to TV medium enabled *lrgAB* to be induced at stationary phase, suggesting that saliva could provide a favorable environment for pyruvate uptake through LrgAB. In this study, we used commercial pooled saliva, collected from multiple donors and realized that the growth was poor and inconsistent when the cell was grown in saliva alone, depending the batches. High viscosity of saliva could be another factor that interferes with the GFP quantification assay. Therefore, in this study, we diluted saliva with TV broth that lacks substance(s) required for P*lrgA* activation. Our data provides insight for the functionality of LrgAB as a pyruvate uptake system *in vivo*, i.e., in the oral environment.

From a metabolic standpoint, the excretion of pyruvate can occur when production of pyruvate exceeds the organism’s capacity to metabolize pyruvate during growth. Since pyruvate stands at a major metabolic node in aerobic and anaerobic metabolism, it is not surprising that the expression of genes encoding the enzymes for the catabolic pathways of pyruvate, is also altered in accordance with the expression of *lrgAB*. The fact that expression of *pdhC* and *pfl* (responsible for the conversion of pyruvate to acetyl-CoA in the presence and absence of oxygen, respectively), known to be upregulated at stationary phase compared to exponential phase, was not altered during the transition to stationary phase in TV11, suggests that tryptone may provide an unfavorable metabolic condition for the conversion of glucose to pyruvate (glycolysis), consequently accumulating pyruvate and subsequently decreasing the intracellular level of acetyl-CoA in the cell. Given the upregulation of *pta* (responsible for the conversion of acetyl-CoA to acetyl-P) at stationary phase, it is also possible that the level of acetyl-P may be correlated with *lrg* expression, which is currently under investigation. Interestingly, glycolysis is also reported to be affected by K^+^ concentrations in eukaryotic cells (Eckel et al., 1966; Olerud et al., 1975; Peng et al., 1994). In bacteria, the uptake of sugars, amino acids, and other nutrients would occur with the regulation of the cytoplasmic concentration of K^+^ and other ions (Kakinuma, 1998). *S. mutans* is often exposed to severe conditions in which a K^+^ circulation cannot satisfy their requirements in key metabolic fluxes. Thus, further study of the linkage between K^+^ uptake and metabolic fluxes, including pyruvate and glycolysis, could be an interesting topic in future.

The results presented herein also suggest that constitutive expression of LrgAB guarantees pyruvate uptake sufficient for exponential growth in TV, regardless of acetate or K^+^, but is unable to get over the stationary-phase repression of P*lrgA*. These findings indicate that the effects of acetate, K^+^, and pyruvate specifically exert their LrgAB regulatory properties during the transition to stationary phase, requiring a complex network of metabolic adaptations that are, at least in part, coordinated by LytST in response to environmental signals. Sensing environmental acetate, K^+^, and pyruvate by the LytST-LrgAB circuit ensures an optimization of the cell’s physiological state for metabolic homeostasis. Cellular decisions for pyruvate flux also seem to rapidly and sensitively be made in response to both the external and internal metabolic status, due to the fast-turnover nature of pyruvate at stationary phase. In conclusion, this study reveals a previously unacknowledged and important role for environmental acetate and K^+^ in controlling pyruvate uptake through LrgAB. Acetate and/or K^+^ may function as metabolic and/or environmental signals, allowing cells to more efficiently sense an increase in external pyruvate level before this increase becomes subjected to feedback regulation. Furthermore, given the hypothetical role of LrgAB in inducing cell death and lysis, it also seems plausible that these factors may also be involved in eliciting cell death and lysis. Considering that the concentration of acetate and K^+^ would be very different in the media, extensively used for *S. mutans* study, and is abundant in dental plaque fluids, we will need to use extra caution in choosing the growth media, and designing *in vivo* experiments, especially if the study under question is related to metabolic components and their regulation.

## Data Availability Statement

All datasets generated for this study are included in the article/Supplementary Material.

## Author Contributions

S-JA contributed to conception, design, acquisition, analysis, and interpretation, and drafted the manuscript. SD, LB, and ML contributed to acquisition, analysis, and interpretation. KR contributed to conception and interpretation and helped to draft and edit the manuscript. All authors gave the final approval and agreed to be accountable for all aspects of the work.

## Conflict of Interest

The authors declare that the research was conducted in the absence of any commercial or financial relationships that could be construed as a potential conflict of interest.

## Footnotes

1 https://www.bdbiosciences.com/documents/bionutrients_tech_manual.pdf

## References

[B1] Abbe K. Carlsson J. Takahashi-Abbe S. Yamada T. (1991). Oxygen and the sugar metabolism in oral streptococci. *Proc. Finn. Dent. Soc.* 87 477–487. 1775476

[B2] Ahn S. J. Deep K. Turner M. E. Ishkov I. Waters A. Hagen S. J. (2019). Characterization of LrgAB as a stationary phase-specific pyruvate uptake system in *Streptococcus mutans*. *BMC Microbiol.* 19:223. 10.1186/s12866-019-1600-x 31606034 PMC6790026

[B3] Ahn S. J. Qu M. D. Roberts E. Burne R. A. Rice K. C. (2012). Identification of the *Streptococcus mutans* LytST two-component regulon reveals its contribution to oxidative stress tolerance. *BMC Microbiol.* 12:187. 10.1186/1471-2180-12-187 22937869 PMC3507848

[B4] Ahn S. J. Rice K. C. (2016). Understanding the *Streptococcus mutans* Cid/Lrg System through CidB function. *Appl. Environ. Microbiol.* 82 6189–6203. 10.1128/AEM.01499-16 27520814 PMC5068163

[B5] Ahn S. J. Rice K. C. Oleas J. Bayles K. W. Burne R. A. (2010). The *Streptococcus mutans* Cid and Lrg systems modulate virulence traits in response to multiple environmental signals. *Microbiology* 156(Pt 10), 3136–3147. 10.1099/mic.0.039586-0 20671018 PMC3068699

[B6] Axe D. D. Bailey J. E. (1995). Transport of lactate and acetate through the energized cytoplasmic membrane of *Escherichia coli*. *Biotechnol. Bioeng.* 47 8–19. 10.1002/bit.260470103 18623362

[B7] Binepal G. Gill K. Crowley P. Cordova M. Brady L. J. Senadheera D. B. (2016). Trk2 potassium transport system in *Streptococcus mutans* and its role in potassium homeostasis, biofilm formation, and stress tolerance. *J. Bacteriol.* 198 1087–1100. 10.1128/JB.00813-15 26811321 PMC4800877

[B8] Burne R. A. Schilling K. Bowen W. H. Yasbin R. E. (1987). Expression, purification, and characterization of an exo-beta-D-fructosidase of *Streptococcus mutans*. *J. Bacteriol.* 169 4507–4517. 10.1128/jb.169.10.4507-4517.1987 3308844 PMC213815

[B9] Burne R. A. Wen Z. T. Chen Y. Y. Penders J. E. (1999). Regulation of expression of the fructan hydrolase gene of *Streptococcus mutans* GS-5 by induction and carbon catabolite repression. *J. Bacteriol.* 181 2863–2871. 10.1128/jb.181.9.2863-2871.1999 10217779 PMC93730

[B10] Busuioc M. Buttaro B. A. Piggot P. J. (2010). The pdh operon is expressed in a subpopulation of stationary-phase bacteria and is important for survival of sugar-starved *Streptococcus mutans*. *J. Bacteriol.* 192 4395–4402. 10.1128/JB.00574-510 20581205 PMC2937364

[B11] Carlsson J. Kujala U. Edlund M. B. (1985). Pyruvate dehydrogenase activity in *Streptococcus mutans*. *Infect. Immun.* 49 674–678. 10.1128/iai.49.3.674-678.1985 4030096 PMC261240

[B12] Castano-Cerezo S. Bernal V. Post H. Fuhrer T. Cappadona S. Sanchez-Diaz N. C. (2014). Protein acetylation affects acetate metabolism, motility and acid stress response in *Escherichia coli*. *Mol. Syst. Biol.* 10:762. 10.15252/msb.20145227 25518064 PMC4299603

[B13] Charbonnier T. Le Coq D. McGovern S. Calabre M. Delumeau O. Aymerich S. (2017). Molecular and physiological logics of the pyruvate-induced response of a novel transporter in bacillus subtilis. *MBio* 8 e976–17. 10.1128/mBio.00976-17 28974613 PMC5626966

[B14] Chaudhari S. S. Thomas V. C. Sadykov M. R. Bose J. L. Ahn D. J. Zimmerman M. C. (2016). The LysR-type transcriptional regulator, CidR, regulates stationary phase cell death in Staphylococcus aureus. *Mol. Microbiol.* 101 942–953. 10.1111/mmi.13433 27253847 PMC5014633

[B15] Christian J. H. Waltho J. A. (1961). The sodium and potassium content of non-halophilic bacteria in relation to salt tolerance. *J. Gen. Microbiol.* 25 97–102. 10.1099/00221287-25-1-97 13693390

[B16] Colby S. M. Russell R. R. (1997). Sugar metabolism by mutans streptococci. *Soc. Appl. Bacteriol. Symp. Ser.* 26 80S–88S. 10.1046/j.1365-2672.83.s1.9.x 9436320

[B17] Constantopoulos G. Barranger J. A. (1984). Nonenzymatic decarboxylation of pyruvate. *Anal. Biochem.* 139 353–358. 10.1016/0003-2697(84)90016-2 6476373

[B18] Csonka L. N. (1989). Physiological and genetic responses of bacteria to osmotic stress. *Microbiol. Rev.* 53 121–147. 10.1128/mmbr.53.1.121-147.1989 2651863 PMC372720

[B19] Dashper S. G. Reynolds E. C. (1992). pH regulation by *Streptococcus mutans*. *J. Dent. Res.* 71 1159–1165. 10.1177/00220345920710050601 1607433

[B20] Datta A. (1991). Characterization of the inhibition of *Escherichia coli* pyruvate dehydrogenase complex by pyruvate. *Biochem. Biophys. Res. Commun.* 176 517–521. 10.1016/0006-291x(91)90955-7 2018539

[B21] Desagher S. Glowinski J. Premont J. (1997). Pyruvate protects neurons against hydrogen peroxide-induced toxicity. *J. Neurosci.* 17 9060–9067. 10.1523/jneurosci.17-23-09060.1997 9364052 PMC6573585

[B22] Dibdin G. H. Shellis R. P. Dawes C. (1986). A comparison of the potassium content and osmolality of plaque fluid and saliva, and the effects of plaque storage. *J. Dent. Res.* 65 1053–1056. 10.1177/00220345860650080301 3461021

[B23] Eckel R. E. Rizzo S. C. Lodish H. Berggren A. B. (1966). Potassium transport and control of glycolysis in human erythrocytes. *Am. J. Physiol.* 210 737–743. 10.1152/ajplegacy.1966.210.4.737 4222110

[B24] Fox D. K. Meadow N. D. Roseman S. (1986). Phosphate transfer between acetate kinase and enzyme I of the bacterial phosphotransferase system. *J. Biol. Chem.* 261 13498–13503. 3020035

[B25] Gawron K. Wojtowicz W. Lazarz-Bartyzel K. Lamasz A. Qasem B. Mydel P. (2019). Metabolomic status of the oral cavity in chronic periodontitis. *In Vivo* 33 1165–1174. 10.21873/invivo.11587 31280206 PMC6689338

[B26] Giandomenico A. R. Cerniglia G. E. Biaglow J. E. Stevens C. W. Koch C. J. (1997). The importance of sodium pyruvate in assessing damage produced by hydrogen peroxide. *Free Radic Biol. Med.* 23 426–434. 10.1016/s0891-5849(97)00113-5 9214579

[B27] Gueriri I. Bay S. Dubrac S. Cyncynatus C. Msadek T. (2008). The Pta-AckA pathway controlling acetyl phosphate levels and the phosphorylation state of the DegU orphan response regulator both play a role in regulating Listeria monocytogenes motility and chemotaxis. *Mol. Microbiol.* 70 1342–1357. 10.1111/j.1365-2958.2008.06496.x 19019159

[B28] Hansen H. G. Henning U. (1966). Regulation of pyruvate dehydrogenase activity in *Escherichia coli* K12. *Biochim. Biophys. Acta* 122 355–358. 10.1016/0926-6593(66)90076-2 4291045

[B29] Hawver L. A. Giulietti J. M. Baleja J. D. Ng W. L. (2016). Quorum sensing coordinates cooperative expression of pyruvate metabolism genes to maintain a sustainable environment for population stability. *MBio* 7 e1863–6. 10.1128/mBio.01863-16 27923919 PMC5142617

[B30] Jolkver E. Emer D. Ballan S. Kramer R. Eikmanns B. J. Marin K. (2009). Identification and characterization of a bacterial transport system for the uptake of pyruvate, propionate, and acetate in Corynebacterium glutamicum. *J. Bacteriol.* 191 940–948. 10.1128/JB.01155-08 19028892 PMC2632059

[B31] Kakinuma Y. (1998). Inorganic cation transport and energy transduction in *Enterococcus* hirae and other streptococci. *Microbiol. Mol. Biol. Rev.* 62 1021–1045. 10.1128/mmbr.62.4.1021-1045.1998 9841664 PMC98938

[B32] Kaspar J. Shields R. C. Burne R. A. (2018). Competence inhibition by the XrpA peptide encoded within the comX gene of *Streptococcus mutans*. *Mol. Microbiol.* 109 345–364. 10.1111/mmi.13989 29802741 PMC6158096

[B33] Kaspar J. Underhill S. A. M. Shields R. C. Reyes A. Rosenzweig S. Hagen S. J. (2017). Intercellular communication via the comX-Inducing Peptide (XIP) of *Streptococcus mutans*. *J. Bacteriol.* 199 e404–17. 10.1128/JB.00404-17 28808131 PMC5626963

[B34] Kim H. M. Waters A. Turner M. E. Rice K. C. Ahn S. J. (2018). Regulation of cid and lrg expression by CcpA in *Streptococcus mutans*. *Microbiology* 165 113–123. 10.1099/mic.0.000744 30475201 PMC6600348

[B35] Kim S. B. Shin B. S. Choi S. K. Kim C. K. Park S. H. (2001). Involvement of acetyl phosphate in the in vivo activation of the response regulator ComA in *Bacillus subtilis*. *FEMS Microbiol. Lett.* 195 179–183. 10.1111/j.1574-6968.2001.tb10518.x 11179649

[B36] Kim S. K. Wilmes-Riesenberg M. R. Wanner B. L. (1996). Involvement of the sensor kinase EnvZ in the in vivo activation of the response-regulator PhoB by acetyl phosphate. *Mol. Microbiol.* 22 135–147. 10.1111/j.1365-2958.1996.tb02663.x 8899716

[B37] Kuhn M. L. Zemaitaitis B. Hu L. I. Sahu A. Sorensen D. Minasov G. (2014). Structural, kinetic and proteomic characterization of acetyl phosphate-dependent bacterial protein acetylation. *PLoS One* 9:e94816. 10.1371/journal.pone.0094816 24756028 PMC3995681

[B38] Lauderdale K. J. Malone C. L. Boles B. R. Morcuende J. Horswill A. R. (2010). Biofilm dispersal of community-associated methicillin-resistant *Staphylococcus* aureus on orthopedic implant material. *J. Orthop. Res.* 28 55–61. 10.1002/jor.20943 19610092

[B39] Liu X. Pena Sandoval G. R. Wanner B. L. Jung W. S. Georgellis D. Kwon O. (2009). Evidence against the physiological role of acetyl phosphate in the phosphorylation of the ArcA response regulator in *Escherichia coli*. *J. Microbiol.* 47 657–662. 10.1007/s12275-009-0087-9 19851741

[B40] Margolis H. C. Moreno E. C. (1994). Composition and cariogenic potential of dental plaque fluid. *Crit. Rev. Oral. Biol. Med.* 5 1–25. 10.1177/10454411940050010101 7999948

[B41] Mizunoe Y. Wai S. N. Takade A. Yoshida S. (1999). Restoration of culturability of starvation-stressed and low-temperature-stressed *Escherichia coli* O157 cells by using H2O2-degrading compounds. *Arch. Microbiol.* 172 63–67. 10.1007/s002030050741 10398754

[B42] O’Donnell-Tormey J. Nathan C. F. Lanks K. DeBoer C. J. de la Harpe J. (1987). Secretion of pyruvate. An antioxidant defense of mammalian cells. *J, Exp. Med.* 165 500–514. 10.1084/jem.165.2.500 3102672 PMC2188509

[B43] Olerud J. E. Pryor W. H. Jr. Eason R. L. Carroll H. W. (1975). The role of potassium ion in muscle glycogenolysis and glycolysis. *Proc. Soc. Exp. Biol. Med.* 150 677–680. 10.3181/00379727-150-39104 1208589

[B44] Paczia N. Nilgen A. Lehmann T. Gatgens J. Wiechert W. Noack S. (2012). Extensive exometabolome analysis reveals extended overflow metabolism in various microorganisms. *Microb. Cell. Fact.* 11 122. 10.1186/1475-2859-11-122 22963408 PMC3526501

[B45] Peng L. Zhang X. Hertz L. (1994). High extracellular potassium concentrations stimulate oxidative metabolism in a glutamatergic neuronal culture and glycolysis in cultured astrocytes but have no stimulatory effect in a GABAergic neuronal culture. *Brain Res.* 663 168–172. 10.1016/0006-8993(94)90475-8 7850466

[B46] Pinhal S. Ropers D. Geiselmann J. de Jong H. (2019). Acetate metabolism and the inhibition of bacterial growth by acetate. *J. Bacteriol.* 201 147–166. 10.1128/JB.00147-19 30988035 PMC6560135

[B47] Rice K. C. Nelson J. B. Patton T. G. Yang S. J. Bayles K. W. (2005). Acetic acid induces expression of the Staphylococcus aureus cidABC and lrgAB murein hydrolase regulator operons. *J. Bacteriol.* 187 813–821. 10.1128/JB.187.3.813-821.2005 15659658 PMC545714

[B48] Rice K. C. Turner M. E. Carney O. V. Gu T. Ahn S. J. (2017). Modification of the *Streptococcus mutans* transcriptome by LrgAB and environmental stressors. *Microb Genom.* 3:e000104. 10.1099/mgen.0.000104 28348880 PMC5361627

[B49] Sadykov M. R. Thomas V. C. Marshall D. D. Wenstrom C. J. Moormeier D. E. Widhelm T. J. (2013). Inactivation of the Pta-AckA pathway causes cell death in *Staphylococcus aureus*. *J. Bacteriol.* 195 3035–3044. 10.1128/JB.00042-13 23625849 PMC3697545

[B50] Sato Y. Noji S. Suzuki R. Taniguchi S. (1989). Dual mechanism for stimulation of glutamate transport by potassium ions in *Streptococcus mutans*. *J. Bacteriol.* 171 4963–4966. 10.1128/jb.171.9.4963-4966.1989 2768193 PMC210304

[B51] Schwartz E. R. Reed L. J. (1970). Regulation of the activity of the pyruvate dehydrogenase complex of *Escherichia coli*. *Biochemistry* 9 1434–1439. 10.1021/bi00808a019 4907331

[B52] Shen L. C. Atkinson D. E. (1970). Regulation of pyruvate dehydrogenase from *Escherichia coli*. Interactions of adenylate energy charge and other regulatory parameters. *J. Biol. Chem.* 245 5974–5978. 4320794

[B53] Smirnova G. V. Oktiabr’skii O. N. (1988). Effect of the activity of primary proton pumps on the growth of *Escherichia coli* in the presence of acetate. *Mikrobiologiia* 57 554–559. 2905416

[B54] Son M. Ahn S. J. Guo Q. Burne R. A. Hagen S. J. (2012). Microfluidic study of competence regulation in *Streptococcus mutans*: environmental inputs modulate bimodal and unimodal expression of comX. *Mol. Microbiol.* 86 258–272. 10.1111/j.1365-2958.2012.08187.x 22845615 PMC3468698

[B55] Son M. Ghoreishi D. Ahn S. J. Burne R. A. Hagen S. J. (2015). Sharply tuned pH response of genetic competence regulation in *Streptococcus mutans*: a microfluidic study of the environmental sensitivity of comX. *Appl. Environ. Microbiol.* 81 5622–5631. 10.1128/AEM.01421-15 26070670 PMC4510173

[B56] Takahashi N. Washio J. Mayanagi G. (2010). Metabolomics of supragingival plaque and oral bacteria. *J. Dent. Res.* 89 1383–1388. 10.1177/0022034510377792 20924070

[B57] Terleckyj B. Shockman G. D. (1975). Amino acid requirements of *Streptococcus mutans* and other oral streptococci. *Infect. Immun.* 11 656–664. 10.1128/iai.11.4.656-664.1975 1091547 PMC415118

[B58] Terleckyj B. Willett N. P. Shockman G. D. (1975). Growth of several cariogenic strains of oral streptococci in a chemically defined medium. *Infect. Immun.* 11 649–655. 10.1128/iai.11.4.649-655.1975 1091546 PMC415117

[B59] Thomas V. C. Sadykov M. R. Chaudhari S. S. Jones J. Endres J. L. Widhelm T. J. (2014). A central role for carbon-overflow pathways in the modulation of bacterial cell death. *PLoS Pathog.* 10:e1004205. 10.1371/journal.ppat.1004205 24945831 PMC4063974

[B60] van den Esker M. H. Kovacs A. T. Kuipers O. P. (2017). YsbA and LytST are essential for pyruvate utilization in *Bacillus subtilis*. *Environ Microbiol.* 19 83–94. 10.1111/1462-2920.13454 27422364

[B61] Vilhena C. Kaganovitch E. Grunberger A. Motz M. Forne I. Kohlheyer D. (2019). Importance of pyruvate sensing and transport for the resuscitation of viable but nonculturable *Escherichia coli* K-12. *J. Bacteriol.* 201 e610–18. 10.1128/JB.00610-18 30420452 PMC6349089

[B62] Vilhena C. Kaganovitch E. Shin J. Y. Grunberger A. Behr S. Kristoficova I. (2018). A single-cell view of the BtsSR/YpdAB pyruvate sensing network in *Escherichia coli* and its biological relevance. *J. Bacteriol.* 200 e536–17. 10.1128/JB.00536-17 29038258 PMC5717152

