# Supplementary material for: Acetate and Potassium Modulate the Stationary-Phase Activation of lrgAB in Streptococcus mutans
Source: Front Microbiol. 2020 Mar 13;11:401. doi: 10.3389/fmicb.2020.00401 (PMC7082836; doi:10.3389/fmicb.2020.00401)
Supplement: Supplementary file 1 [file Data_Sheet_1.docx]

Supplementary Material

Supplementary Figure 1. Measurement of extracellular pyruvate during growth of S. mutans wild-type strain in BHI.

**Supplementary Figure 2.** Change of P*lrgA* activity during growth in T11 and TVY11 media, supplemented by 0 (A and E), 1 (B and F), 10 (C and G), and 40 mM (D and H) pyruvate (pyr).

**Supplementary Figure 3.** The effect of exogenously added pyruvate on the growth of the *lrgAB*-overexpressing strain in BHI.

**Supplementary Figure 4.** Change of pH over growth of *S. mutans* wild type strain in FMC11 (A), BHI (B), TV11 (C), TY11 (D), and kTV (E) media.

**Supplementary Figure 5.** Change of P*lrgA* activity during growth in kTV11 and nTV11 media, supplemented by 0 (A and E), 1 (B and F), 10 (C and G), and 40 mM (D and H) of extracellular pyruvate (pyr).

**Supplementary Figure 6.** The effect of exogenously added pyruvate on the growth of *S. mutans* wild type strain in kTV11 (A) and nTV11 (B) media.

**Supplementary Figure 7.** Effect of KCl on expression of *lrgA* at stationary phase by real-time qPCR.

**Supplementary Figure 8.** The effect of different concentrations (A, 10 mM; B, 20 mM; C, 50 mM, and D, 100 mM) of potassium, supplemented as K-phosphate, on eliciting the P*lrgA* activation in FMC11**.**

**Supplementary Figure 9.** Identification of acetate and potassium as medium components, allowing P*lrgA* activation in TV11, a non-preferred medium for stationary-phase *lrg* induction.
